# Safety and efficacy of long‐term use of a buprenorphine transdermal patch system in patients with osteoarthritis and low back pain refractory to non‐opioid analgesics: Post‐marketing surveillance of 3000 cases

**DOI:** 10.1111/papr.13430

**Published:** 2024-10-21

**Authors:** Takahiro Ushida, Rumiko Kanzaki, Keishi Katayama, Akito Ishikawa

**Affiliations:** ^1^ Aichi Medical University Nagakute‐shi Aichi Japan; ^2^ Scientific Affairs Division Mundipharma K.K. Minato‐ku, Tokyo Japan

**Keywords:** buprenorphine transdermal patch system, chronic pain, post‐marketing surveillance

## Abstract

**Objective**s**:**

A post‐marketing surveillance was conducted to evaluate the safety and efficacy of the buprenorphine transdermal patch under actual clinical practice.

**Results:**

Of the 3017 patients included in the safety analysis, adverse drug reactions (ADRs) were observed in 1524 (50.5%), the most common being nausea, skin symptoms at the site of application, constipation, and vomiting. The incidences of respiratory depression and withdrawal symptoms were low, and no drug dependence was observed. Among the 2573 patients included in the efficacy analysis, the efficacy (≥2‐point improvement in the numerical rating scale) rate was 74.4%, which was significantly higher in older adults (≥65 y.o) than in younger adults. Discontinuation was mostly caused by ADRs during the early initiation phase.

**Conclusion:**

This study demonstrated the safety and efficacy of long‐term administration of buprenorphine transdermal patches, suggesting that pain control is possible over the long term if attention is paid to ADRs in the early stages of administration.

## INTRODUCTION

Chronic pain is defined as pain that persists for more than 3 months or beyond the usual treatment period.^1^ The prevalence of chronic pain in Japan is estimated to be 15%–39%.^2^, ^3^, ^4^ Non‐steroidal anti‐inflammatory drugs (NSAIDs), acetaminophen, and opioid analgesics are often used to treat chronic non‐cancerous musculoskeletal pain, which is the main cause of chronic pain.^1^

Opioids exert analgesic effects by acting on opioid receptors distributed mainly in the nervous system and by inhibiting nociceptive transmission at brain, spinal cord and peripheral nervous systems.^5^ Opioids have been recognized to be effective for various types of chronic pain,^6^, ^7^ but owing to adverse drug reactions (ADRs) and addiction risks specific to opioids,^6^, ^7^, ^8^ various guidelines indicate that opioids should be used when non‐opioid drugs are ineffective.^1^, ^5^, ^9^ A buprenorphine transdermal patch system (BTDS) (NORSPAN® TAPE) is a transdermal long‐acting pain treatment agent applied once a week, containing buprenorphine as an active ingredient. It was approved in Japan in February 2011 for the treatment of chronic pain associated with osteoarthritis and low back pain that is refractory to non‐opioid analgesics. Buprenorphine has been shown to have fewer respiratory ADRs than other μ opioids^10^, ^11^ and a lower risk of drug misuse.^10^, ^12^ Buprenorphine is primarily excreted in the feces, with low risk of accumulation in patients with impaired renal function or older adults.^11^, ^13^, ^14^

We conducted post‐marketing surveillance to evaluate the safety and efficacy of BTDS in real‐world clinical practice.

## METHODS

### Design

The protocol for this study was approved by the Pharmaceuticals and Medical Devices Agency and was conducted in accordance with Good Post‐Marketing Study Practice (GPSP) in Japan.

This survey aimed to investigate the safety and efficacy of BTDS during long‐term use in clinical practice and focus on opioid‐specific ADRs such as nausea, vomiting, and skin symptoms at the site of application. This multicenter prospective survey, with central registration and an observation period of 52 weeks, was conducted between August 2011 and July 2016. The target number of patients was set at 3000, as the number of patients in which unknown ADRs occurred at a frequency of 0.1% could be detected with a probability of at least 95%.

### Patients and methods

BTDS is indicated in Japan for patients with osteoarthritis (OA) or low back pain who have difficulty with activities of daily living that do not respond adequately to conservative therapy, including non‐opioid analgesics. Cases of pain related to OA or low back pain refractory to non‐opioid analgesics and regarded as chronic state were eligible for this survey and enrolled between August 2011 and July 2015. The BTDS is a matrix‐type transdermal formulation containing buprenorphine and is available in three dosage forms, each containing 5, 10, or 20 mg of buprenorphine (In Japan, the dose of BTDS is expressed as the amount of contained, not the release rate). BTDS was applied to the anterior chest, upper back, outer upper arm, or lateral chest according to the package insert and was replaced every 7 days. BTDS was started at 5 mg and subsequently adjusted according to symptoms, with the maximum amount applied being less than 20 mg.

### Endpoints

Safety was evaluated in accordance with MedDRA/J version 21.1 for ADRs including serious ADRs in the safety analysis population. Serious adverse events were defined as those that corresponded to the following: death, life‐threatening, results in permanent disability or a congenital anomaly, leads to or prolongs a hospital admission, and medically significant. Nausea, vomiting, dizziness, somnolence, constipation, respiratory depression, weight loss, risk of QTc interval prolongation, skin symptoms at the site of application, withdrawal symptoms, and drug dependence were defined as priority surveillance ADRs. Nausea includes nausea, abnormal feeling, chest discomfort, abdominal discomfort, and loss of appetite, because the reporters reported these events as nausea related events or symptoms. Incidence of priority surveillance ADRs was evaluated according to sex and age. The association between antiemetic use and ADRs was also investigated.

Efficacy was evaluated using the numerical rating scale (NRS) for pain intensity in the efficacy analysis population. A reduction of 2 points or more was defined as effective in the NRS, which consists of 11 levels, and the effective rate was calculated. NRS for OA and low back pain were calculated over time and at last measurements. The last measurement was defined as the last measurement during the period in which the BTDS was administered to the case. Treatment goals set by the patients regarding sleep, exercise, and work/housework were evaluated on a four‐point scale: achieved, almost achieved, approximately half achieved, and not very achieved. The sum of the achieved and almost‐achieved was calculated as the treatment goal achievement rate. For patients who continued the BTDS for 52 weeks, changes in the NRS and treatment goal achievement rate were calculated for each number of the priority surveillance ADRs.

### Statistics

The safety analysis population comprised patients who received a BTDS applied at least once. Among the safety analysis population, patients in whom the efficacy of the BTDS was evaluated at least once were included in the efficacy analysis population. Descriptive statistics were expressed as *n* (%), mean ± SD, and median [range]. The chi‐square test was used to compare categorical variables, the Cochran‐Armitage trend test was used to evaluate the association of ordinal scale data, and the paired t‐test was used to compare the NRS scores before and after treatment. The last observation carried forward method was used to impute the missing values in the NRS.

## RESULTS

### Patient characteristics

**FIGURE 1:**
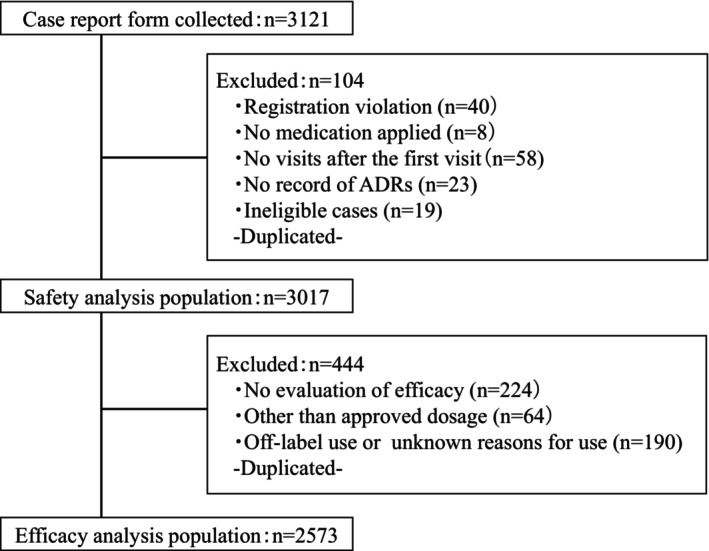
Patient flow. ADRs, Adverse drug reactions.

Case report forms for 3121 patients were collected, and 3017 were included in the safety analysis. One hundred and four cases were excluded due to registration violations or non‐administration (Figure 1). A total of 2573 patients were included in the efficacy analysis after excluding 444 patients from the safety analysis population, such as those with no efficacy evaluation. The safety analysis population consisted of 68.2% of females, with a mean age of 73.0 years, 80.3% older adults (≥65 y.o), and a mean NRS of 7.7 (Table 1). There were 2238 patients (74.2%) with complications and 2756 (91.3%) with concomitant medication.

**TABLE 1:** Patient characteristics.

*n*	3017
Sex (female), *n* (%)	2059 (68.2%)
Age (years)	
Mean ± SD	73.0 ± 12.45
Median [range]	76.0 [18–101]
<65 years old, *n* (%)	594 (19.7%)
≥65 years old, *n* (%)	2423 (80.3%)
Height (cm), mean ± SD	154.0 ± 9.9
Weight (kg), mean ± SD	56.1 ± 12.1
Clinical department, *n* (%)	
Orthopedic surgery	2303 (76.3%)
Anesthesiology	220 (7.3%)
Pain clinic	35 (1.2%)
Surgery	154 (5.1%)
Internal medicine	250 (8.3%)
Other	55 (1.8%)
Primary disease, *n* (%)	
Osteoarthritis	1003 (33.2%)
Low back pain	1824 (60.5%)
Other	190 (6.3%)
Duration of chronic pain (month), mean ± SD	50.9 ± 61.0
NRS	
Mean ± SD	7.7 ± 1.5
Median [range]	8.0 [2–10]
Hypersensitivity predisposition, *n* (%)	
No	2911 (96.5%)
Yes	96 (3.2%)
Unknown	10 (0.3%)
Medical history, *n* (%)	
No	2131 (70.6%)
Yes	886 (29.4%)
Complications, *n* (%)	
No	779 (25.8%)
Yes	2238 (74.2%)
Liver disease^a^	41 (1.4%)
Kidney disease^b^	128 (4.2%)
Dialysis	26 (0.9%)
Switching from pre‐administered analgesics, *n* (%)	
No	2165 (71.8%)
Yes	846 (28.0%)
Taking analgesics within 1 week after switching	79 (2.6%)
Unknown	6 (0.2%)
Concomitant medications, *n* (%)	
No	261 (8.7%)
Yes	2756 (91.3%)
Opioid^c^	110 (3.6%)
NSAIDs^c^	1718 (56.9%)

Abbreviation: NSAIDs, non‐steroidal anti‐inflammatory drugs.

^a^ Liver disease included autoimmune hepatitis, cholelithiasis, chronic hepatitis, alcoholic cirrhosis, cirrhosis, hepatic cysts, abnormal hepatic function, fatty liver, hepatitis, alcoholic hepatitis, hepatic dysfunction, gallbladder polyp, cholecystectasia, hepatitis B, hepatitis C, liver abscess, hepatitis virus carrier, hepatitis C virus test positive, malignant neoplasm of the liver, and hepatic encephalopathy.

^b^ Kidney disease included renal anemia, renal edema, pyelonephritis, kidney cancer, ureteral stones, urinary tract lithiasis, difficulty urinating, chronic glomerulonephritis, glucosuria, tonic bladder, nephrolithiasis, nephropathy, nephrotic syndrome, neurogenic bladder, nocturia, frequent micturition, renal aneurysm, kidney cyst, renal disorder, renal failure, chronic renal failure, renal hypertension, cystocele, diabetic nephropathy, renal dysfunction, erosive cystitis, stress incontinence, hemodialysis, kidney transplantation, and dialysis.

^c^ For opioids and NASAIDs, the proportion of patients receiving each drug among those receiving concomitant medications was calculated.

### ADRs

ADRs were observed in 1524 patients (50.5%) (Table 2). The most common symptom was nausea in 704 patients (23.3%), followed by constipation in 371 (12.3%) and vomiting in 315 (10.4%). Serious ADRs were observed in 73 patients (1.7%), the most common being nausea in 14 (0.5%) and vomiting in 13 (0.4%). The incidence of ADRs according to patient background was significantly lower in males (44.6% vs. 53.3%, *p* < 0.001) and older adult patients (49.5% vs. 54.7%, *p* = 0.024), and significantly higher in patients with complications (53.8% vs. 41.0%, p < 0.001) (Table 3). The incidence of ADRs by complications was 53.7%, 45.3%, and 50.0% in patients with liver disease, renal disease, and dialysis, respectively. Among the 11 priority surveillance ADRs, the most common were nausea (including abdominal discomfort, chest discomfort, loss of appetite, and abnormal sensations) in 711 patients (23.6%), followed by skin symptoms at the application site in 519 (17.2%), constipation in 371 (12.3%), and vomiting in 315 (10.4%) (Table 4). Respiratory depression was observed in 26 patients (0.9%). Withdrawal symptoms were observed in 2 patients (0.1%), and there were no cases of drug dependence.

**TABLE 2:** ADRs: Incidence ≥1%.

*N* = 3017	Severity		
	Serious	Non‐serious	Total
Incidence rate, *n* (%)	51 (1.7%)	1473 (48.8%)	1524 (50.5%)
Number of occurrences	73	2719	2795
Incidence rate by each ADR, *n* (%)			
Decreased appetite	2 (0.1%)	59 (2.0%)	61 (2.0%)
Somnolence	–	210 (7.0%)	210 (7.0%)
Dizziness	3 (0.1%)	198 (6.6%)	201 (6.7%)
Nausea	14 (0.5%)	690 (22.9%)	704 (23.3%)
Constipation	1 (0.0%)	370 (12.3%)	371 (12.3%)
Vomiting	13 (0.4%)	302 (10.0%)	315 (10.4%)
Contact dermatitis	1 (0.0%)	139 (4.6%)	140 (4.6%)
Pruritus at the site of application	1 (0.0%)	192 (6.4%)	193 (6.4%)
Indicated site erythema	–	61 (2.0%)	61 (2.0%)
Indicated site dermatitis	–	125 (4.1%)	125 (4.1%)
Weight loss	1 (0.0%)	57 (1.9%)	58 (1.9%)

*Note*: ADRs were summarized based on MedDRA/J version 21.1.

Abbreviation: ADRs, adverse drug reactions.

**TABLE 3:** Incidence of ADRs and efficacy rate by patient background.

	Incidence of ADRs (*n* = 3017)	*p*	Efficacy rate (*n* = 2544)	*p*
Incidence of ADRs and efficacy rate, *n* (%)	1524 (50.5%)		1893 (74.4%)	
Sex, *n* (%)				
Male	427 (44.6%)	*p* < 0.001^a^	578 (73.0%)	*p* = 0.266^a^
Female	1097 (53.3%)		1315 (75.1%)	
Age class, *n* (%)				
<65 years old	325 (54.7%)	*p* = 0.024^a^	304 (70.0%)	*p* = 0.022^a^
≥65 years old	1199 (49.5%)		1589 (75.3%)	
Weight (kg)				
<40 kg	68 (47.9%)	*p* = 0.907^b^	86 (71.1%)	*p* = 0.657^b^
≥40 to <50 kg	333 (49.8%)		432 (76.7%)	
≥50 to <60 kg	468 (56.3%)		525 (73.8%)	
≥60 to <70 kg	260 (44.8%)		357 (73.0%)	
≥70 kg	186 (53.1%)		210 (74.7%)	
Unknown	209 (47.0%)		283 (74.7%)	
Primary disease, *n* (%)				
Osteoarthritis	517 (51.6%)	*p* = 0.012^a^	683 (75.3%)	*p* = 1.000^a^
Knee	430 (50.5%)		575 (75.0%)	
Groin	56 (57.7%)		68 (77.3%)	
Elbow	9 (64.3%)		8 (72.7%)	
Other	52 (55.9%)		66 (73.3%)	
Low back pain	894 (49.0%)		1210 (73.9%)	
Duration of chronic pain, *n* (%)				
<1 month	5 (41.7%)	*p* < 0.001^b^	10 (90.9%)	*p* = 0.004^b^
≥1 to <3 months	71 (32.4%)		143 (82.7%)	
≥3 to <6 months	131 (52.4%)		144 (75.0%)	
≥6 to <12 months	124 (46.8%)		161 (74.5%)	
≥12 to <36 months	368 (48.8%)		478 (74.5%)	
≥36 to <120 months	549 (54.0%)		646 (73.4%)	
≥120 months	228 (57.3%)		244 (70.3%)	
NRS, *n* (%)				
0	0	*p* < 0.001^b^	0	*p* < 0.001^b^
1	0		0	
2	5 (71.4%)		0	
3	19 (55.9%)		10 (35.7%)	
4	36 (80.0%)		20 (55.6%)	
5	109 (53.4%)		97 (56.1%)	
6	122 (51.9%)		131 (66.2%)	
7	341 (55.0%)		365 (68.4%)	
8	483 (47.6%)		686 (78.0%)	
9	228 (47.7%)		354 (83.1%)	
10	153 (49.5%)		230 (87.1%)	
Complications, *n* (%)				
No	319 (41.0%)	*p* < 0.001^a^	474 (74.4%)	*p* = 1.000^a^
Yes	1205 (53.8%)		1419 (74.4%)	
Liver disease^c^	22 (53.7%)		27 (77.1%)	
Kidney disease^d^	58 (45.3%)		88 (80.0%)	
Dialysis	13 (50.0%)		17 (77.3%)	
Switching from pre‐administered analgesics, *n* (%)				
No	1113 (51.4%)	*p* = 0.138^a^	1341 (73.4%)	*p* = 0.082^a^
Yes	409 (48.4%)		549 (76.8%)	
Taking analgesics within 1 week after switching	37 (46.8%)		60 (82.2%)	

Abbreviations: ADRs, adverse drug reactions; NRS, numerical rating scale.

^a^ Chi‐square test.

^b^ Cochran‐Armitage trend test.

^c^ Liver disease included autoimmune hepatitis, cholelithiasis, chronic hepatitis, alcoholic cirrhosis, cirrhosis, hepatic cysts, abnormal hepatic function, fatty liver, hepatitis, alcoholic hepatitis, hepatic dysfunction, gallbladder polyp, cholecystectasia, hepatitis B, hepatitis C, liver abscess, hepatitis virus carrier, hepatitis C virus test positive, malignant neoplasm of the liver, and hepatic encephalopathy.

^d^ Kidney disease included renal anemia, renal edema, pyelonephritis, kidney cancer, ureteral stones, urinary tract lithiasis, difficulty urinating, chronic glomerulonephritis, glucosuria, tonic bladder, nephrolithiasis, nephropathy, nephrotic syndrome, neurogenic bladder, nocturia, frequent micturition, renal aneurysm, kidney cyst, renal disorder, renal failure, chronic renal failure, renal hypertension, cystocele, diabetic nephropathy, renal dysfunction, erosive cystitis, stress incontinence, hemodialysis, kidney transplantation, and dialysis.

**TABLE 4:** Time of first occurrence of priority surveillance ADRs.

	Total	Time of first occurrence (days)								
		1–3	4–7	8–14	15–21	22–28	29–56	57–84	85‐	Unknown
	*N* = 3017	*N* = 3017	*N* = 2913	*N* = 2778	*N* = 2532	*N* = 2290	*N* = 2138	*N* = 1655	*N* = 1384	
Priority surveillance ADRs										
Nausea^a^	711 (23.6%)	249 (8.3%)	78 (2.7%)	96 (3.5%)	91 (3.6%)	36 (1.6%)	86 (4.0%)	35 (2.1%)	37 (2.7%)	3
Vomiting	315 (10.4%)	117 (3.9%)	34 (1.2%)	46 (1.7%)	34 (1.3%)	18 (0.8%)	35 (1.6%)	15 (0.9%)	15 (1.1%)	1
Dizziness	201 (6.7%)	56 (1.9%)	25 (0.9%)	30 (1.1%)	22 (0.9%)	16 (0.7%)	28 (1.3%)	9 (0.5%)	15 (1.1%)	
Somnolence	210 (7.0%)	51 (1.7%)	29 (1.0%)	44 (1.6%)	27 (1.1%)	16 (0.7%)	29 (1.4%)	9 (0.5%)	5 (0.4%)	
Constipation	371 (12.3%)	53 (1.8%)	57 (2.0%)	73 (2.6%)	52 (2.1%)	37 (1.6%)	62 (2.9%)	26 (1.6%)	10 (0.7%)	1
Respiratory depression	26 (0.9%)	9 (0.3%)	2 (0.1%)	1 (0.0%)	4 (0.2%)	1 (0.0%)	5 (0.2%)	1 (0.1%)	3 (0.2%)	
Weight loss	58 (1.9%)			1 (0.0%)	2 (0.1%)	4 (0.2%)	12 (0.6%)	14 (0.9%)	24 (1.7%)	1
Risk of QTc interval prolongation	8 (0.3%)		1 (0.0%)		1 (0.0%)	1 (0.0%)	2 (0.1%)		1 (0.1%)	2
Skin symptoms at the application site	519 (17.2%)	38 (1.3%)	43 (1.5%)	71 (2.6%)	52 (2.1%)	31 (1.4%)	95 (4.4%)	73 (4.4%)	114 (8.2%)	2
Withdrawal symptoms	2 (0.1%)				1 (0.0%)					1
Drug dependence	0									

Abbreviation: ADRs, adverse drug reactions.

^a^ Including nausea, abnormal feeling, chest discomfort, abdominal discomfort, and loss of appetite.

### Dosing status

Of the 3017 patients included in the safety analysis, 590 (19.6%) continued BTDS for 52 weeks. A total of 2427 patients (80.4%) discontinued BTDS within 51 weeks, and 36.1% of patients discontinued BTDS within 28 days. The most common reason for discontinuation was ADRs in 1029 patients (42.4%), followed by pain improvement in 483 patients (19.9%), and insufficient effect in 340 patients (14.0%). The ADRs leading to discontinuation included nausea in 419 patients (16.9%), vomiting in 218 (8.8%), and dizziness in 132 (5.3%).

### Nausea and vomiting

Among the priority surveillance ADRs, the most common was nausea in 711 patients (23.6%). Of the 3017 patients included in the safety analysis, 996 (33.0%) received antiemetic drugs to prevent nausea and vomiting. The incidence of nausea and vomiting was also higher in the group that received antiemetic prophylaxis (34.7% with antiemetics vs. 20.1% without antiemetics) (data not shown). Nausea and vomiting occurred frequently up to the third day after treatment initiation (Table 4) and were more frequent in females (Table 5). There was no difference in the incidence of nausea and vomiting between older and younger adults. Of the 753 patients who developed nausea/vomiting, 297 continued BTDS even after the onset of these symptoms, of whom 288 patients (96.9%) recovered or improved, and the median time to recovery/improved was 8.0 days (range: 1–190 days) (data not shown). Antiemetics were administered to treat nausea and vomiting in 182 of the 288 patients (61.3%); however, there was no clear difference in the rate of recovery/remission (96.2% with antiemetics vs. 98.3% with no‐antiemetics) or the median time to recovery (8.0 days vs. 8.0 days), depending on whether antiemetics were administered. Metoclopramide (86 patients) was the most used antiemetic, followed by domperidone (60 patients) and prochlorperazine (41 patients).

**TABLE 5:** Occurrence of priority surveillance ADRs by sex and age.

	Total (*n* = 3017)	Sex		Age class	
		Male (*n* = 958)	Female (*n* = 2059)	>65 years old (*n* = 594)	≥65 years old (*n* = 2423)
Incidence of ADRs, *n* (%)	1524 (50.5%)	427 (44.6%)	1097 (53.3%)	325 (54.7%)	1199 (49.5%)
Incidence of priority surveillance ADRs, *n* (%)					
Nausea^a^	711 (23.6%)	179 (18.7%)	532 (25.8%)	152 (25.6%)	559 (23.1%)
Vomiting	315 (10.4%)	65 (6.8%)	250 (12.1%)	65 (10.9%)	250 (10.3%)
Dizziness	201 (6.7%)	53 (5.5%)	148 (7.2%)	42 (7.1%)	159 (6.6%)
Somnolence	210 (7.0%)	61 (6.4%)	149 (7.2%)	72 (12.1%)	138 (5.7%)
Constipation	371 (12.3%)	136 (14.2%)	235 (11.4%)	72 (12.1%)	299 (12.3%)
Respiratory depression	26 (0.9%)	6 (0.6%)	20 (1.0%)	7 (1.2%)	19 (0.8%)
Weight loss	58 (1.9%)	19 (2.0%)	39 (1.9%)	13 (2.2%)	45 (1.9%)
Risk of QTc interval prolongation	8 (0.3%)	4 (0.4%)	4 (0.2%)	2 (0.3%)	6 (0.3%)
Skin symptoms at the application site	519 (17.2%)	148 (15.5%)	371 (18.0%)	150 (25.3%)	369 (15.2%)
Withdrawal symptoms	2 (0.1%)	0	2 (0.1%)	0	2 (0.1%)
Drug dependence	0	0	0	0	0

Abbreviation: ADRs, adverse drug reactions.

^a^ Including nausea, abnormal feeling, chest discomfort, abdominal discomfort, and loss of appetite.

### Efficacy

**FIGURE 2:**
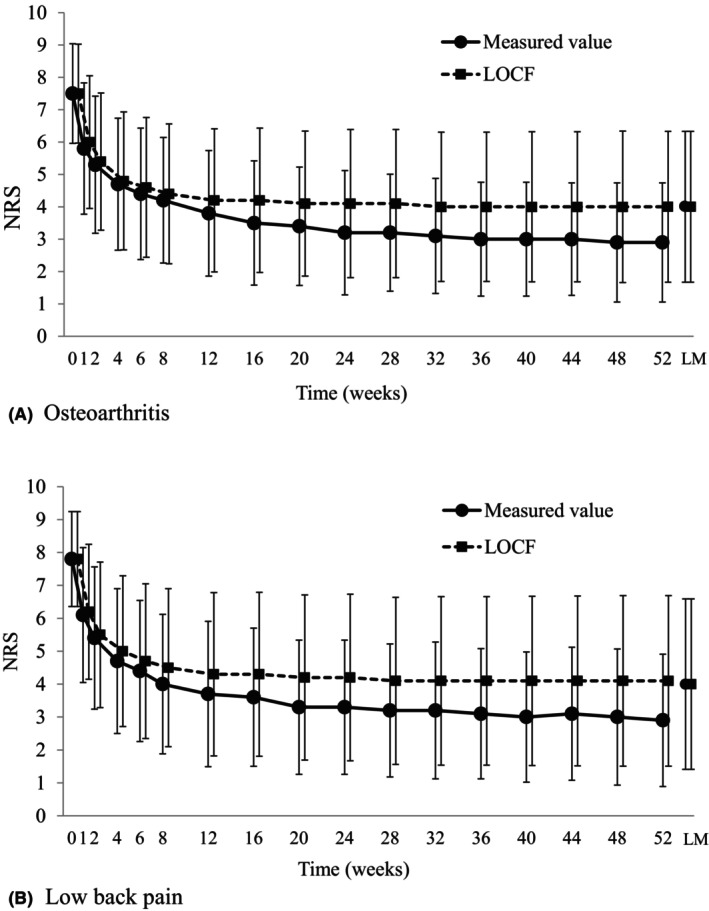
Change in NRS: Efficacy analysis population. LM, Last measurement; LOCF, Last observation carried forward; NRS, Numerical rating scale. (A) In patients with osteoarthritis, measured NRS significantly decreased from 7.5 ± 1.5 at baseline to 2.9 ± 1.8 at 52 weeks (*p* < 0.001) and 4.0 ± 2.3 at last measurement (*p* < 0.001). In the LOCF analysis, NRS significantly decreased from 7.5 ± 1.5 at baseline to 4.0 ± 2.3 at 52 weeks (*p* < 0.001) and 4.0 ± 2.3 at last measurement (*p* < 0.001). (B) In patients with low back pain, measured NRS significantly decreased from 7.8 ± 1.4 at baseline to 2.9 ± 2.0 at 52 weeks (*p* < 0.001) and 4.0 ± 2.6 at last measurement (*p* < 0.001). In the LOCF analysis, NRS significantly decreased from 7.8 ± 1.4 at baseline to 4.1 ± 2.6 at 52 weeks (*p* < 0.001) and 4.0 ± 2.6 at last measurement (*p* < 0.001).

NRS in patients with OA was 7.5 ± 1.5 at baseline, 2.9 ± 1.8 at 52 weeks, and 4.0 ± 2.3 at last measurement, with changes of −4.8 ± 2.2 (*p* < 0.001) and − 3.6 ± 2.6 (*p* < 0.001), respectively (Figure 2A). Similarly, in patients with low back pain, NRS was 7.8 ± 1.4 at baseline, 2.9 ± 2.0 at week 52, and 4.0 ± 2.6 at last measurement, with changes of −4.8 ± 2.5 (*p* < 0.001) and − 3.7 ± 2.8 (*p* < 0.001), respectively (Figure 2B). The efficacy rate was 74.4%, which was significantly higher in older adults than in younger adults (75.3% vs. 70.0%, *p* = 0.022) and higher in those with higher baseline NRS (*p* < 0.001) (Table 3).

### Achievement rates of treatment goals

The achievement rates of the treatment goals for sleep set by the patients were 53.9% at 4 weeks, 71.8% at 12 weeks, 74.9% at 24 weeks, 82.9% at 52 weeks, and 67.7% at last measurement. Similarly, for exercise, it was 34.4% at 4 weeks, 55.3% at 12 weeks, 59.9% at 24 weeks, 70.1% at 52 weeks, and 53.1% at last measurement. Regarding work/housework, 38.9% at 4 weeks, 57.8% at 12 weeks, 65.7% at 24 weeks, 75.8% at 52 weeks, and 55.7% at last measurement.

### Relationship between safety and efficacy

Table 6 shows the NRS change and achievement rates of treatment goals according to the number of priority surveillance ADRs (0, 1–2, and ≥3) in 551 patients of 2573 patients in the efficacy analysis population who continued to use BTDS for 52 weeks. Of the 551 patients, 411 (74.6%) did not experience any of the priority surveillance ADRs, with a change in NRS of −5.00 ± 2.28 at 52 weeks and an achievement rate of treatment goal of 86.1% for sleep, 74.7% for exercise, and 78.3% for work/housework. There were 121 patients (22.0%) who experienced one or two priority surveillance ADRs, with a change in NRS of −4.23 ± 2.45 at 52 weeks, and achievement rate of treatment goal of 76.1% for sleep, 59.6% for exercise, and 67.5% for work/housework. Nineteen patients (3.5%) experienced three or more priority surveillance ADRs, with a change in NRS of −3.18 ± 2.65 at 52 weeks and achievement rates of 44.4% for sleep, 35.7% for exercise, and 75.0% for work/housework, respectively. As the number of ADRs increased, the achievement rate of treatment goals decreased.

**TABLE 6:** NRS change and achievement rates of treatment goal according to the number of priority surveillance ADRs in patients who continued to buprenorphine transdermal patch for 52 weeks.

	Total (*N* = 2573)	Patients who continued buprenorphine transdermal patch for 52 weeks (*n* = 551)					
		Partial total (*n* = 551)	Change in NRS at 52 weeks (*n* = 509)		Achievement rate of treatment goals at 52 weeks		
					Sleep (*n* = 332)	Exercise (*n* = 399)	Work/housework (*n* = 320)
	*n* (%)	*n* (%)	*n*	Mean ± SD	*n*/*N* (%)	*n*/*N* (%)	*n*/*N* (%)
Number of priority surveillance ADRs							
0	1402 (54.5%)	411 (74.6%)	384	−5.00 ± 2.28	217/252 (86.1%)	221/296 (74.7%)	184/235 (78.3%)
1–2	1004 (39.0%)	121 (22.0%)	108	−4.23 ± 2.45	54/71 (76.1%)	53/89 (59.6%)	52/77 (67.5%)
≧3	167 (6.5%)	19 (3.5%)	17	−3.18 ± 2.65	4/9 (44.4%)	5/14 (35.7%)	6/8 (75.0)%

*Note*: In the efficacy analysis population, the change in NRS and the achievement rate of treatment goals were calculated by the number of priority ADRs for 551 patients who continued buprenorphine transdermal patch for 52 weeks.

Abbreviations: ADRs, adverse drug reactions; NRS, numerical rating scale.

## DISCUSSION

This is the first report on post‐marketing surveillance of BTDS in Japanese patients with non‐cancer chronic pain. The study population consisted of 68.2% women, with a mean age of 73.0 years, a mean duration of chronic pain of 50.9 months (4.3 years), and a mean NRS of 7.7. Inoue et al.^4^ reported that the mean age of patients with chronic pain in Japan was 60.9 years, 60.9% were female, and the mean NRS was 5.2. In post‐marketing surveillance of an oral tramadol/acetaminophen combination drug in 1262 patients with non‐cancer pain refractory to non‐opioid analgesics conducted by Yoshizawa et al.,^15^ the mean age was 66.8 years, 61.3% were female, the mean duration of chronic pain was 2.0 years, and the mean NRS was 7.1. Our study had a high proportion of women, which is similar to epidemiological and previous studies using other opioids; however, our study population was relatively older, had a longer disease duration, and had a higher NRS. ADRs were observed in 50.5% of the patients, and the incidence was higher in females than in males (53.3% vs. 44.6%). It has been suggested that women are more responsive to opioids than men,^16^, ^17^ and similar results were observed in our study.

Of the 3017 patients included in the safety analysis, 590 (19.6%) continued treatment with BTDS for 52 weeks. The most common reason for discontinuation of treatment was ADRs in 1029 patients (42.4%), followed by pain improvement in 483 (19.9%), insufficient effect in 340 (14.0%), and no show‐up in 266 (11.0%). The discontinuation rates were 29.0% at 4 weeks and 53.9% at 12 weeks. Generally, the rate of continued administration of opioid analgesics for non‐cancer pain is not high.^18^ Of 279 patients who experienced ADRs due to transdermal fentanyl for non‐cancer pain, 173 (62.0%) discontinued treatment.^19^ In addition, the post‐marketing surveillance of the tramadol/acetaminophen combination drug for non‐cancer pain by Yoshizawa et al.^15^ also showed that the median time from initiation of treatment to discontinuation was 23 days, with discontinuation rates of 20.9% at 4 weeks and 45.2% at 12 weeks. The treatment continuation status in our study was similar to those of previous studies.

The incidence of ADRs was relatively higher in patients with complications (53.8% vs. 41.0%), but the incidence in patients with renal disease and undergoing dialysis was 45.3% and 50.0%, respectively, which was not significantly different from the overall incidence (50.5%). These results are consistent with the pharmacokinetic property that buprenorphine is primarily excreted in the feces. In addition, the incidence of ADRs in patients with liver disease was 53.7%, which was not significantly different from the overall incidence. Buprenorphine has been shown to increase its bioavailability in severe hepatic failure, but does not affect clearance in mild to moderate hepatic dysfunction.^20^ Buprenorphine is metabolized in the liver, which is estimated to account for approximately 1/3 of all buprenorphine.^21^ This study also suggests that the effect of hepatic function on clearance may be relatively small.

In our study, the change in NRS using BTDS in patients with OA and low back pain was −3.5 and −3.8, respectively, which was almost equivalent to the results of post‐marketing surveillance of tramadol/acetaminophen combination drug in patients with non‐cancer pain unrelieved by non‐opioid drugs by Yoshizawa et al. (change in NRS: −3.43).^15^ In our study, achievement rates of treatment goals related to sleep, exercise, and work/housework were higher, with greater reductions in NRS (data not shown), suggesting that pain reduction with BTDS may have contributed to the improvement of patient's quality of life.

The relationship between the number of priority surveillance ADRs and efficacy was analyzed in 551 patients treated with BTDS for 52 weeks. This analysis showed that if treatment was continued after the onset of priority surveillance ADRs, the rate of achievement of treatment goals for sleep, exercise, and work increased. It was also shown that as the number of ADRs increased, the rate of achievement of treatment goals decreased. Nausea (23.6%) was the most common priority surveillance ADR and the most common cause of discontinuation (16.9%), suggesting that prevention of nausea and vomiting is important for the continued use of BTDS.

The Japanese guidelines for prescribing opioid analgesics for chronic non‐cancer chronic pain recommend prophylactic antiemetic administration when necessary.^5^ However, the incidence of nausea/vomiting in our study showed no effect of the prophylactic administration of antiemetic agents (34.7% with antiemetics vs. 20.1% without antiemetics). One possible reason for this is that patients prescribed prophylactic antiemetics may be more aware of nausea than necessary because of the nocebo effect. Therefore, careful attention should be paid to the use of antiemetic agents to prevent nausea and vomiting.

Although the incidence of nausea/vomiting was relatively high from the start of treatment to the third day (nausea: 35.0% and vomiting: 37.1%), most patients who continued using BTDS, even after the onset of these symptoms, recovered or improved (median time to recovered/improved: 8 days). This result was similar to a report^1^, ^22^ that nausea and vomiting caused by opioids tend to occur at the beginning of administration or when the dose is increased, and that with continued administration, tolerance develops and the symptoms disappear or are reduced. Therefore, regarding nausea and vomiting, conditions of the patients should be carefully monitored at the beginning of treatment to determine the continuation of the treatment.

This study had several limitations. The safety analysis population in this study was likely to be older and had higher NRS than the general population with chronic pain. The efficacy rate evaluation using the NRS was a univariate analysis, and other influencing factors were not considered. The results of this study need to be interpreted with these considerations in mind.

## CONCLUSION

This study demonstrated the safety and efficacy of BTDS for long‐term use. BTDS may cause ADRs early after treatment initiation; however, in cases where treatment can be continued, they have been shown to have long‐term efficacy for non‐cancer chronic pain, even in older adults and patients with renal dysfunction.

## AUTHOR CONTRIBUTIONS

All authors participated in the interpretation of the study results and in the drafting, critical revision, and approval of the final version of the manuscript. RK was involved in the study design and study management, TU provided medical adviser in the study. AI conducted the statistical analysis.

## FUNDING INFORMATION

This survey was conducted by Mundipharma K.K. as a post‐marketing surveillance.

## CONFLICT OF INTEREST STATEMENT

Takahiro Ushida received consulting fees from Mundipharma K.K. Rumiko Kanzaki, Keishi Katayama, and Akito Ishikawa are employees of Mundipharma K.K.

## PATIENT CONSENT

Patient consent was not mandatory as this study was conducted in accordance with Good Post‐Marketing Study Practice (GPSP) by the Ministry of Health, Labour and Welfare in Japan.

## Data Availability

Data from this study will not be shared.

## References

[1] Working Group for the Development of Clinical Practice Guidelines for the Management of Chronic Pain . Clinical practice guidelines for the management of chronic pain. Tokyo, Japanese: Shinko Trading Company Ltd., Publication Department of Medical Books; 2021.

[2] Nakamura M , Toyama Y , Nishiwaki Y , Ushida T . Prevalence and characteristics of chronic musculoskeletal pain in Japan. J Orthop Sci. 2011;16:424–432. 21678085 10.1007/s00776-011-0102-y PMC3140943

[3] Takura T , Ushida T , Kanchiku T , Ebata N , Fujii K , DiBonaventura MC , et al. The societal burden of chronic pain in Japan: an internet survey. J Orthop Sci. 2015;20:750–760. 25963609 10.1007/s00776-015-0730-8

[4] Inoue S , Kobayashi F , Nishihara M , Arai YCP , Ikemoto T , Kawai T , et al. Chronic pain in the Japanese community—prevalence, characteristics and impact on quality of life. PLoS One. 2015;10:e0129262. 26076135 10.1371/journal.pone.0129262 PMC4467865

[5] Japan Society of Pain Clinicians . Guidelines for Prescribing Opioid Analgesics for Chronic Non‐cancer pain. Japanese. https://www.jspc.gr.jp/Contents/public/kaiin_guideline04.html

[6] Furlan AD , Sandoval JA , Mailis‐Gagnon A , Tunks E . Opioids for chronic noncancer pain: a meta‐analysis of effectiveness and side effects. CMAJ. 2006;174:1589–1594. 16717269 10.1503/cmaj.051528 PMC1459894

[7] Kalso E , Edwards JE , Moore AR , McQuay HJ . Opioids in chronic non‐cancer pain: systematic review of efficacy and safety. Pain. 2004;112:372–380. 15561393 10.1016/j.pain.2004.09.019

[8] Ling W , Mooney L , Hillhouse M . Prescription opioid abuse, pain and addiction: clinical issues and implications. Drug Alcohol Rev. 2011;30:300–305. 21545561 10.1111/j.1465-3362.2010.00271.x PMC4170948

[9] Tsumura H . OARSI recommendations for the Management of Knee Osteoarthritis: evidence‐based expert consensus guidelines from OARSI. J Jpn Soc Int Med. 2017;106:75–83.

[10] Dahan A , Yassen A , Romberg R , Sarton E , Teppema L , Olofsen E , et al. Buprenorphine induces ceiling in respiratory depression but not in analgesia. Br J Anaesth. 2006;96:627–632. 16547090 10.1093/bja/ael051

[11] Pergolizzi J , Aloisi AM , Dahan A , Filitz J , Langford R , Likar R , et al. Current knowledge of buprenorphine and its unique pharmacological profile. Pain Pract. 2010;10:428–450. 20492579 10.1111/j.1533-2500.2010.00378.x

[12] Brennan MJ . Update on prescription extended‐release opioids and appropriate patient selection. J Multidiscip Healthc. 2013;6:265–280. 23900563 10.2147/JMDH.S38562 PMC3726523

[13] al‐Tawil N , Odar‐Cederlöf I , Berggren AC , Johnson HE , Persson J . Pharmacokinetics of transdermal buprenorphine patch in the elderly. Eur J Clin Pharmacol. 2013;69:143–149. 22706617 10.1007/s00228-012-1320-8 PMC3548110

[14] Pergolizzi J , Böger RH , Budd K , Dahan A , Erdine S , Hans G , et al. Opioids and the management of chronic severe pain in the elderly: consensus statement of an international expert panel with focus on the six clinically most often used World Health Organization step III opioids (buprenorphine, fentanyl, hydromorphone, methadone, morphine, oxycodone). Pain Pract. 2008;8:287–313. 18503626 10.1111/j.1533-2500.2008.00204.x

[15] Yoshizawa K , Kawai K , Fujie M , Suzuki J , Ogawa Y , Yajima T , et al. Overall safety profile and effectiveness of tramadol hydrochloride/acetaminophen in patients with chronic noncancer pain in Japanese real‐world practice. Curr Med Res Opin. 2015;31:2119–2129. 26359328 10.1185/03007995.2015.1091975

[16] Whitley H , Lindsey W . Sex‐based differences in drug activity. Am Fam Physician. 2009;80:1254–1258. 19961138

[17] Bartley EJ , Fillingim RB . Sex differences in pain: a brief review of clinical and experimental findings. Br J Anaesth. 2013;111:52–58. 23794645 10.1093/bja/aet127 PMC3690315

[18] Deyo RA , Von Korff M , Duhrkoop D . Opioids for low back pain. BMJ. 2015;350:g6380. 25561513 10.1136/bmj.g6380 PMC6882374

[19] Lee J , Yoon JS , Lee JH , Chung SH , Lee KY , Kim YY , et al. Clinical usefulness of long‐term application of fentanyl matrix in chronic non‐cancer pain: improvement of pain and physical and emotional functions. Clin Orthop Surg. 2016;8:465–474. 27904731 10.4055/cios.2016.8.4.465 PMC5114261

[20] Davis MP , Pasternak G , Behm B . Treating chronic pain: an overview of clinical studies centered on the buprenorphine option. Drugs. 2018;78:1211–1228. 30051169 10.1007/s40265-018-0953-z PMC6822392

[21] Przeklasa‐Muszyńska A , Dobrogowski J . Transdermal buprenorphine in the treatment of cancer and non‐cancer pain – the results of multicenter studies in Poland. Pharmacol Rep. 2011;63:935–948. 22001981 10.1016/s1734-1140(11)70609-0

[22] Ferrarese F , Becchimanzi G , Bernardo M , Conte MA , Gioia A , Ottaviani D , et al. Pain treatment with high‐dose, controlled‐release oxycodone: an Italian perspective. Ther Clin Risk Manag. 2008;4:665–672. 19209246 PMC2621386

